# Drug safety of rosiglitazone and pioglitazone in France: a study using the French PharmacoVigilance database

**DOI:** 10.1186/1472-6904-11-5

**Published:** 2011-05-24

**Authors:** Stephanie Berthet, Pascale Olivier, Jean-Louis Montastruc, Maryse Lapeyre-Mestre

**Affiliations:** 1 Unité INSERM 1027, Equipe de Pharmacoépidémiologie, Université de Toulouse (Université Paul Sabatier), Toulouse, France; 2 Centre Régional de Pharmacovigilance, de Pharmacoépidémiologie et d'Information sur le Médicament, Service de Pharmacologie Clinique, Hôpitaux de Toulouse, Toulouse, France

## Abstract

**Background:**

Thiazolidinediones (TZDs), rosiglitazone (RGZ) and pioglitazone (PGZ) are widely used as hypoglycemic drugs in patients with type 2 diabetes mellitus. The aim of our study was to investigate the profile of adverse drug reactions (ADRs) related to TZDs and to investigate potential risk factors of these ADRs.

**Methods:**

Type 2 diabetic patients were identified from the French Database of PharmacoVigilance (FPVD) between 2002 and 2006. We investigated ADR related to TZD, focusing on 4 ADR: edema, heart failure, myocardial infarction and hepatitis corresponding to specific WHO-ART terms.

**Results:**

Among a total of 99,284 adult patients in the FPVD, 2295 reports concerned type 2 diabetic patients (2.3% of the whole database), with 161 (7%) exposed to TZDs. The frequency of edema and cardiac failure was significantly higher with TZDs than in other patients (18% and 7.4% versus 0.8% and 0.1% respectively, p < 0.001) whereas the frequency of hepatitis was similar (5.9% versus 4%, NS). A multiple logistic regression model taking into account potential confounding factors (age, gender, drug exposure and co-morbidities) found that TZD exposure remained associated with heart failure and edema, but not with hepatitis or myocardial infarction.

**Conclusions:**

Thiazolidinediones exposure is associated with an increased risk of edema and heart failure in patients with type 2 diabetes even when recommendations for use are respected. In contrast, the risk of hepatic reactions and myocardial infarction with this class of drugs seems to be similar to other hypoglycemic agents.

## Background

Thiazolidinediones (TZDs) are peroxisome proliferator-activated receptor (PPAR) agonists which regulate transcription of genes encoding proteins involved in glucose and lipid metabolism. Troglitazone, the first agent of this class, caused serious liver toxicity leading to its withdrawal in 2000, less than 3 years after its marketing [1]. The use of the 2 other TZDs, rosiglitazone (RGZ) and pioglitazone (PGZ), has sharply increased during the last few years. These 2 drugs seem to present a lower risk of hepatotoxicity than troglitazone [2].

TZDs could also induce adverse drug reactions (ADRs) related to the cardiovascular system including edema and heart failure [3,4]. Edema is more frequent when the TZD is used in combination therapy and its incidence is higher in association with insulin. [4]. Because of the risk of congestive heart failure [5], the use of RGZ and PGZ was initially contraindicated in France in patients with a cardiac insufficiency corresponding to classes I to IV of the NYHA classification. The European Medicines Agency recommended the suspension of marketing authorizations for rosiglitazone-containing anti-diabetes medicines in Europe in September 2010 [6]. This decision followed the publication of 2 studies finding an increased cardiovascular risk of rosiglitazone [7,8]. In view of the restrictions already in place on the use of rosiglitazone in Europe, no additional measures have been identified that could reduce this cardiovascular risk.

The aim of our study was to investigate the profile of adverse drug reactions (ADRs) related to TZDs as reported to the French PharmacoVigilance System in type 2 diabetic patients, with a special focus on congestive heart failure and myocardial infarction, and to investigate factors associated with these ADRs.

## Methods

We used the data from the French national system of PharmacoVigilance, which has been described before [9,10]. All suspected ADRs are evaluated using a French standardized scale of causality assessment and registered in the French PharmacoVigilance Database (FPVD) [11]. For each report, information on patient's data (age, gender, medical history) and drug exposure (suspected and concomitantly used drugs) is recorded along with a brief clinical description. ADRs are coded according to ADR Terminology of the World Health Organization (WHO-ART) [12].

RGZ was the first TZD marketed at the end of 2001 in France. Therefore we performed searches in the French PharmacoVigilance Database for ADRs reported from January 2002 to December 2006. Among all cases of ADRs reported in the database, patients exposed to drugs approved in the treatment of diabetes in France were short-listed, and we selected only patients with type 2 diabetes. The following data were collected: age, gender, medical history (coded ICD 10^th^), and all drugs (coded according to the ATC classification) used, whether or not they were related to the present ADR. Several co morbidities were identified from medical history and use of drugs. The ADR were described according to the WHO-ART classification and presented as SOC terms. Several WHO-ART codes were retained to specifically describe edema (SOC term cardiovascular disorders and metabolic and nutritional disorders: edema, peripheral edema, low limbs edema), heart failure (SOC term cardiovascular disorders: cardiac failure, congestive cardiac failure, pulmonary edema), myocardial infarction (SOC term cardiovascular disorders and myocardia : myocardial infarction, cardiac death), and hepatitis (SOC term Liver and biliary system disorders: abnormal hepatic functions, abnormal ASAT-ALAT values, hepatitis).

The demographic and clinical characteristics of diabetic patients exposed and non-exposed to TZDs were compared using the *χ*2 test or Fisher's exact test for qualitative variables and using the Student's t-test for quantitative variables. In a further step, association between use of TZDs and occurrence of edema, hepatitis, cardiac failure or myocardial infarction was examined in a bivariate analysis. In order to take into account potential confounding factors (age, gender, cardiovascular co-morbidities and other drugs), a multivariate analysis was performed using a backward logistic regression model. The Hosmer and Lemeshow procedure [13] was used to check the good fitting of the models. All analyses were done with the SAS^® ^software version 9.1.

## Results

Out of 99,284 adult patients registered in the FPVD between January 2002 and December 2006, 2295 were patients with type 2 diabetes (2.3% of the database). Table 1 presents the demographic and clinical characteristics of this population: half of the patients were men; the mean age was 67 (± 13) years, they presented a high frequency of comorbidities, with 27.0% suffering from other metabolic disorders than diabetes, 25.9% with previous heart attack, and 10.8% with cardiac insufficiency. Half of the population was exposed to sulfamides, 40% to metformin and 5% was also treated with insulin. One hundred and sixty-one patients (7%) were exposed to TZDs: 46.6% used pioglitazone, 44.7% used rosiglitazone, and 11.2% used roziglitazone + metformin (4 patients were exposed successively to rosiglitazone alone then to roziglitazone + metformin). These patients were younger, less frequently exposed to sulfamides and glinides, to statins and to NSAIDs than other diabetic patients. They presented less frequently cardiac insufficiency, but they were more frequently obese.

**Table 1:** Demographic and clinical characteristics of patients identified in the French PharmacoVigilance Database with type 2 diabetes.

	*Total population N = 2295 (%)*	*TZD exposed N = 161 (%)*	*TZD not exposed N = 2134 (%)*
**Age **(years)	67.2 (13.4) [19-97]	63.3 (14.2)**** [32-87]	67.5 (13.8) [19-97]
**Gender **(men)	1155 (49.7)	92 (57.1)	1063 (49.8)
**Cardiovascular comorbidities**			
Cardiac arrhythmia	128 (5.58)	8 (5)	120 (5.6)
Cardiac insufficiency	248 (10.81)	9 (5.6)*	239 (*11.2)*
Hypertension	596 (25.97)	33 (*20.5)*	563 (*26.4)*
Metabolic disorders	621 (27.06)	30 (18.7)*	591 (27.7)
Angina pectoris	154 (6.71)	6 (*3.7)*	148 (*6.9)*
Atherosclerosis	6 (0.26)	0 (0)	6 (0.28)
Obesity	76 (3.31)	10 (6.2)*	66 (3.1)
Heart valve disorders	7 (0.31)	1 (0.6)	6 (0.28)
Renal disorders	62 (2.7)	2 (1.2)	60 (2.8)
**Drugs used for type 2 diabetes**			
Sulfamides	1227 (53.46)	44 (27.33)****	1183 (55.43)
Alpha glucosidase inhibitor	207 (9.02)	4 (2.48)**	203 (9.5)
Glinides	192 (8.37)	6 (3.7)*	186 (8.7)
Metformin	900 (39.22)	58(36)	842 (39.5)
Benfluorex	227 (9.89)	4 (2.5)**	223 (10.45)
Insulin	115 (5.01)	4 (2.94)	111 (5.20)
**Cardiovascular drugs**			
Diuretics	627 (27.32)	34 (21.1)	593 (27.7)
ACE inhibitors	456 (19.87)	20 (12.4)*	436 (20.4)
Angiotensin II inhibitors	339 (14.77)	23 (14.3)	316 (14.8)
Digitalics	105 (4.58)	3 (1.9)	102 (4.8)
Betablockers	300 (13.07)	9 (5.6)**	291 (13.6)
Calcium inhibitors	321 (13.99)	16 (9.94)	305 (14.3)
Trinitrin	48 (2.09)	3 (1.9)	45 (2.1)
Amiodarone	103 (4.49)	2 (1.24)*	101 (4.73)
Central antihypertensive drugs	73 (3.18)	5 (3.1)	68 (3.2)
Antiarrythmia drugs	25 (1.09)	1 (0.6)	24 (1.12)
**Other drugs**			
Steroidal and Non Steroidal Anti-Inflammatory Drugs	232 (10.1)	8 (4.96)*	224 (10.5)
Statins	403 (17.56)	17 (10.6)*	386 (16.8)
Fibrates	142 (6.19)	7 (4.35	135 (6.3)

* p < 0.05

** p < 0.01

*** p <0.001

**** p < 0.0001

Quantitative variables are presented as mean (SD), [min-max] and qualitative variables as number (%).

**Figure 1:**
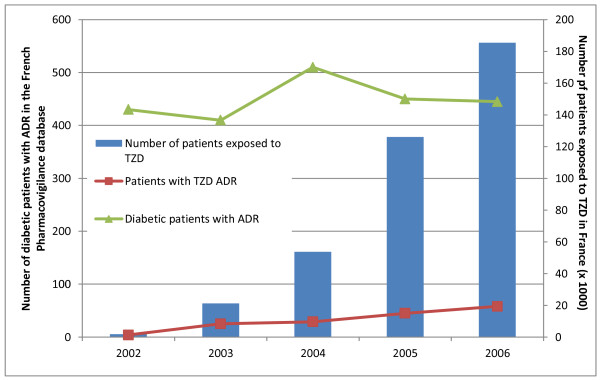
**Evolution of the total number of patients exposed to Thiazolidinediones (TZD) in France and of the total number of Adverse Drug Reactions (ADR) reported to the French PharmacoVigilance Database in type 2 diabetic patients and in TZD exposed patients**.

The number of reports containing ADRs related to TZDs increased from 2002 to the end of 2006, with 4 reports in 2002, 25 in 2003, 29 in 2004, 45 in 2005 and 58 in 2006 (Figure 1). Table 2 presents the most reported ADR which concerned "Body as a whole - general disorders", followed by "Metabolic and nutritional disorders", "Cardiovascular disorders" and "Skin and appendages disorders". Among the 5 ADRs related to sense disorders, 4 were macular edema, and there was no report of fractures. The degree of seriousness of reactions was similar whatever the groups, with 2.53% of ADR leading to death, 6.93% life-threatening, 0.61% leading to sequellae or disability, except for ADRs leading to hospitalization which were less frequent in TZD exposed patients (40.37% versus 53.51%, p < 0.0001) and non-serious ADRs which were more frequent in TZD exposed patients (53.42% versus 36.13%, p < 0.0001).

**Table 2:** Number and percentage of adverse drug reactions reported in the 161 patients exposed to thiazolidinediones in the French PharmacoVigilance database from 2002 to 2006.

System Organ Classification terms	Number of patients (%)
Body as a whole - general disorders 1810	69 (42.8)
Metabolic and nutritional disorders 0800	64 (39.7)
Cardiovascular disorders, general 1010; Myo-, endo-, pericardial & valve disorders 1020; Heart rate and rhythm disorders 1030	42 (26.1)
Skin and appendages disorders 0100	35 (21.7)
Central & peripheral nervous system disorders 0410	33 (20.5)
Red blood cell disorders 1210; White cell and reticulo-endotelial system disorders 1220; Platelet, bleeding & clotting disorders 1230	26 (16.1)
Gastro-intestinal system disorders 0600	21 (13.0)
Liver and biliary system disorders 0700	15 (9.3)
Psychiatric disorders 0500	13 (8.1)
Respiratory system disorders 1100	11 (6.8)
Urinary system disorders 1300	7 (4.3)
Vascular (extra-cardiac) disorders 1040	6 (3.7)
Vision disorders 0431; Hearing and vestibular disorders 0432; Special senses other, disorders 0433	5 (3.1)
Endocrine disorders 0900	4 (2.5)
Immune system	3 (1.9)
Muscular-skeletal system disorders 0200	2 (1.2)
Fetal disorders 1500	2 (1.2)

Total number of adverse drug reactions	358

When considering specific ADR, heart failure was significantly more frequent in TZD patients (5 exposed to RGZ and 7 exposed to PGZ, 7.45% versus 0.14% in non-exposed patients; p < 0.001), as well as edema (9 exposed to RGZ and 20 to PGZ, 18.01% versus 0.84% in non-exposed patients; p < 0.0001). We did not find any difference for hepatitis (4 patients exposed to RGZ and 4 to PGZ 4.97% versus 5.39% in non-exposed patients) and for myocardial infarction (only 1 case exposed to PGZ 0.62% versus 1.18% in non-exposed patients).

Table 3 presents the results of the multivariate analysis concerning the association between TZD exposure and 4 ADRs: heart failure, myocardial infarction, edema and hepatitis. Only edema and heart failure were significantly and independently associated with TZD respectively with an OR of 25.09 and 65.39. Age and obesity were also associated with heart failure, and obesity with edema. Patients treated with biguanides were less exposed to risk of edema. We did not find any association between TZD and myocardial infarction (only heart valve disorders were significantly associated) or hepatitis (as expected, there was a significant association between hepatitis and NSAID).

**Table 3:** Results of the multiple logistic regression models concerning the association between TZD exposure and 4 ADRs: Heart Failure, Myocardial Infarction, Edema and Hepatitis.

Heart failure	Odds ratio	(95% CI)	P
Age	1.04	(0.99 - 1.09)	0.0705
**Obesity**	**5.69**	**(1.34 - 24.20)**	**0.0185**
**Thiazolidinediones**	**65.39**	**(17.678 - 241.93)**	**<0.0001**

*Hosmer and Lemeshow procedure *			*0.32*

**Myocardial infarction**			

Age	0.97	(0.95 - 1.00)	0.1076
Gender	1.73	(0.78 - 3.84)	0.1727
Thiazolidinediones	0.42	(0.05 - 3.17)	0.4039
**Heart valve disorders**	**25.95**	**(2.63 - 255.47)**	**0.0053**
Hypertension	0.34	(0.09 - 1.19)	0.0929

*Hosmer and Lemeshow procedure *			*0.8254*

**Edema**			

Obesity	1.89	(0.58 - 6.17)	0.2863
**Thiazolidinediones**	**25.09**	**(13.50 - 46.63)**	**<0.0001**
Biguanides	0.42	(0.20 - 0.87)	0.02

*Hosmer and Lemeshow procedure *			*0.8043*

**Hepatitis**			

Age	0.99	(0.97 - 1.00)	0.1167
Angina pectoris	0.43	(0.13 - 1.39)	0.1620
Cardiac arrhythmia	0.36	(0.08 - 1.52)	0.1686
Cardiac insufficiency	0.58	(0.25 - 1.37)	0.2217
Thiazolidinediones	0.84	(0.40 - 1.78)	0.6645
**Non Steroidal Anti-Inflammatory Drugs**	**1.85**	**(1.13 - 3.01)**	**0.0134**
Diuretics	0.58	(0.32 - 1.06)	0.0796
Calcium inhibitors	0.63	(0.32 -1.23)	0.1798
Angiotensin II inhibitors	0.68	(0.35 - 1.32)	0.2804

*Hosmer and Lemeshow procedure *			*0.4397*

Potential confounding factors retained in the final model are indicated in the first column.

## Discussion

Among this population of diabetic patients registered in the French Pharmacovigilance database due to the occurrence of an adverse drug reaction, seven percent was exposed to TZDs. In these patients, the reactions were less frequently serious than in patients exposed to other antidiabetic agents. TZD exposure is associated with edema and heart failure in patients with type 2 diabetes, but the risk of hepatic reactions or myocardial infarction with this class of drugs is the same with other hypoglycemic agents.

TZDs can potentially lead to the development of congestive heart failure [1]. In clinical trials, the incidence of edema ranged from 2 to 5% in TZD monotherapy, 6 to 8% with metformin or sulfonylurea and 15% in combination with insulin. In our study, edema represented 18% of ADR related to TZD, and concerned mainly patients exposed to PGZ. Fluid retention can occur even at the lowest TZD dose, and diuretics and ACE inhibitors have variable effects on edema caused by TZDs [1,3].

We found only one case of myocardial infarction in a patient exposed to PGZ, and this event should not be related to this drug. It concerned a 46-year-old man, treated by lamivudine-zidovudine-nevirapine for a HIV infection, and presenting high serum levels of triglycerides 4,57 mmol/l (N< 1,74 mmol/l), cholesterol 8,80 mmol/l (N< 6,56 mmol/l), with a normal value for HDL-cholesterol. He was also treated by glicazide and benfluorex. After one year of treatment with TGZ, he presented a myocardial infarction successfully treated by angioplasty and a drug-eluting stent. The treatment with PGZ was maintained with a favorable evolution. We did not found any other cases with RGZ or PGZ, whereas 26 myocardial infarctions potentially related to drugs were reported in the population of diabetic patients in this study. Two meta-analyses have suggested that RGZ increases the risk of myocardial infarction but did not reach statistical significance for cardiovascular death [14,15], but the reviews published in 2010 led to the rosiglitazone withdrawal from the European market in September 2010. The final results of the RECORD trial, which compared cardiovascular outcomes in patients with type 2 diabetes treated with RGZ and metformin or sulfonylurea, confirm the increasing risk of heart failure with RGZ, but do not identify any statistically significant differences in the overall risk of cardiovascular morbidity or mortality [16]. Lincoff's meta-analysis on the effect of PGZ on ischemic cardiovascular events found that PGZ is associated with a significantly lower risk of death, myocardial infarction, or stroke [17]. In their review of the literature in order to estimate the association between hypoglycemic agents and morbidity and mortality in patients with heart failure and diabetes, Eurich et al [18] concluded that metformin is the only hypoglycemic agent not associated with harm in patients with heart failure. A nested case-control study in older patients found that both PGZ and RGZ were associated with an increased risk of congestive heart failure, acute myocardial infarction, and mortality when compared to other combinations of oral hypoglycemic agents [19].

Cases of hepatotoxicity with second generation TZDs have been few in number and less severe when compared to troglitazone [2,20]. Troglitazone, unlike PGZ and RGZ, induces the cytochrome P450 isoform 3A4, which is partly responsible for its metabolism, and may be prone to drug interactions. Floyd et al examined reports of liver failure reported to the FDA during 10 years and estimated that the rate of acute liver failure observed with RGZ or PGZ was about 17 times higher than the background rate for idiopathic acute liver failure in the general population [21]. By contrast, in our study, we did not find any association between hepatitis and TZD in comparison with other drugs used for diabetes. Even if no reliable estimates of the background rate of liver failure in diabetic patients are available in the literature, some have postulated that liver disease may be more frequent in this population with obesity and insulin resistance, due to non-alcoholic steato-hepatitis [2]. Moreover, this population may be exposed to other drugs, some of which are suspected to increase the risk of hepatic injuries [22], as observed in our data with NSAID.

Some limitations of our study should be discussed. First, limitations are due to the spontaneous reporting system itself, although the reporting rate in France is one of the highest among the European countries [23,24]. Given the small number of patients treated by TZDs in France, the number of ADR reports with TZD is relatively low, in comparison with the results obtained through the Health Canada's spontaneous adverse event reporting system (195 ADR with pioglitazone and 830 with rosiglitazone up to September 2006) [25]. Underreporting can affect validity of results since it can be related either to the drug or to the degree of seriousness of reactions. We did not find any case of myocardial infarction or fracture related to TZD in the database. This is not surprising, since in any spontaneous reporting system, clinicians are unlikely to report this kind of event related to TZD, and instead attribute them to the baseline risk of type 2 diabetes. The absence of fractures reported to the French pharmacovigilance system does not mean that this risk is not real. As demonstrated in the meta-analysis of 10 randomized controlled trials and 2 observational studies, long-term use of TZD doubles the risk of fractures among women with type 2 diabetes, without a significant increase in the risk of fractures in diabetic men [26]. In our study, reported ADRs in the exposed population has increased year by year since the marketing authorization of TZDs in France. This increase can be explained by the increased use of TZDs but also biased by reports related to the notoriety of TZD ADRs. This last point is limited as reports with TZD seem to be less frequently serious than for other diabetic patients.

Populations of patients with specific disease identified through the FPVD are very similar to that obtained through population-based studies in France [27,28]. This population of type 2 diabetic patients with ADR related to their medications presents characteristics comparable to those observed in other studies about French type 2 diabetes [29-31]: for example, we found that 11% of the patients suffered from cardiac insufficiency, which is very similar to the 12% observed in the ENTRED national survey [30]. Moreover, the patterns of exposure to drugs in this population are in agreement with the guidelines for TZD use at the time of the study (in particular, TZDs are contraindicated with insulin and for patients with NYHA class I to IV). In our study, patients exposed to TZDs were less likely to present risk factors of heart failure and cardiovascular comorbidities, 5.6% had a cardiac insufficiency, and less than 3% were treated concomitantly with insulin.

## Conclusions

In the French Pharmacovigilance database, adverse drug reactions reported in diabetic patients exposed to thiazolidinediones (rosiglitazone and pioglitazone) present a degree of seriousness similar to that observed with other anti diabetic drugs. Thiazolidinediones exposure is associated with an increased risk of edema and heart failure in patients with type 2 diabetes even when recommendations for use are respected, and this risk concerns much rosiglitazone as pioglitazone. In contrast, the risk of hepatic reactions and myocardial infarction, which has been discussed with this class of drugs, is not higher than with other hypoglycemic agents.

## Competing interests

None of the authors have any relevant conflict of interest.

SB received a grant from the "Fondation de France" to perform this study during her Master Research training in Clinical Epidemiology (University of Toulouse).

## Authors' contributions

PO and MLM planed the study, SB managed the data, performed the statistical analysis and wrote the main results, JLM and MLM wrote the final manuscript. JLM is the head of the regional pharmacovigilance center and gave his support to access the French pharmacovigilance database. All authors read and approved the final manuscript.

## Pre-publication history

The pre-publication history for this paper can be accessed here:

http://www.biomedcentral.com/1472-6904/11/5/prepub
